# Efficacy of a Four-Week Diagonal Pattern Exercise Program on Trunk and Lower Limb Function in Patients With Stroke-Induced Hemiplegia

**DOI:** 10.7759/cureus.66894

**Published:** 2024-08-14

**Authors:** Bright Alwin Victor, Arunachalam R., Sheela Angel I., Gnanesh Kumar B.

**Affiliations:** 1 Physiotherapy, Madha College of Physiotherapy, Chennai, IND; 2 Neuro Physiotherapy, Madhav University, Pindwara, IND; 3 Neuro Physiotherapy, Vels Institute of Science Technology and Advanced Studies (VISTAS), Chennai, IND

**Keywords:** stroke, diagonal pattern, trunk control, gait, hemiplegia

## Abstract

Objective

The intended effect of this investigation is to quantify the efficacy of a four-week program of diagonal pattern exercises in managing trunk impairments and improving gait difficulties in hemiplegic stroke subjects. The study aims to measure changes in trunk stability and gait parameters post-intervention, providing insights into the potential therapeutic benefits of these exercises for stroke rehabilitation.

Methodology

This experimentation approach encompassing before and the follow-up test evaluations was implemented in this investigation. It was conducted at Madha College of Physiotherapy, Chennai, using convenience sampling to recruit 20 stroke subjects meeting specific inclusion criteria. Participants underwent pre-test evaluations for trunk control and gait. They were then divided equally into two groups for a four-week intervention comprising diagonal pattern exercises or single-plane training. Treatment sessions were administered five days per week for 45 minutes each. Posttest evaluations assessed changes in trunk control using the Trunk Impairment Scale (TIS) and gait parameters via the Timed Up and Go (TUG) test.

Results

Pretest analysis indicated no substantial baseline variations among the experimental and control groups, affirming their suitability for comparison. Posttest analysis of intervention at a 5% significance level revealed that the experimental group demonstrated a statistically significant improvement in trunk control, as measured by the TIS and TUG test, compared to the control group. The paired *t-test* results showed significant differences in pre- and posttest values within each group, while the unpaired *t-*test confirmed the superiority of the experimental group's outcomes, with a *P*-value < 0.05. This improvement is likely due to the effectiveness of the diagonal pattern exercise in enhancing trunk muscle activity and coordination.

Conclusions

This study concludes that diagonal pattern training is more beneficial for improving trunk musculature control and locomotory ability in chronic cerebrovascular accident subjects. The exercise program's simplicity, minimal risks, and ease of home application after initial therapist supervision make it a promising therapeutic approach.

## Introduction

The United Nations World Health Organization assertions that a cerebrovascular accident, or stroke, is an acute medical phenomenon highlighted by the rapidly manifesting neurological symptoms of a focused impairment of brain function that persists for over 24 hours or eventually leads to death with no discernible cause aside from its vascular etiology. The clinical state known as a stroke is represented by the abrupt onset of a sustained confined neurological impairment spurred on following an avascular event. A stroke typically comes accompanied by physical impairments in the individual suffering from it since the event is triggered by a rupture of circulation vessels within the brain or a disruption of the brain's circulatory system [1]. Stroke is mainly classified into two types ischemic and hemorrhagic. Ischemic stroke occurs in 80% of patients and the remaining 20% is hemorrhagic. An artery blockage commonly known as an embolism, an intravascular blood clot, or additional medical conditions that hinder cerebral blood flow may culminate in an ischemic stroke. The destruction of endothelial cells in cerebral blood arteries, reflected in inflammatory and lipid deposition in the intimal layer of the walls of the arteries, is the cause of ischemic stroke. Sixty percent of strokes with ischemic attack victims have not reached the age of 70. Elevated pressure within the cranium damages the brain tissue, and an inhibition in distal blood flow are all associated with hemorrhagic stroke [2]. Globally, stroke is the leading cause of morbidity and mortality rate, stroke is the next in line prominent cause of death after Myocardial infarction [3]. An estimated 12.2 million stroke victims worldwide, 101 million stroke cases are recorded often, and 6.55 million cerebrovascular-related deaths. India has a yearly incidence level of 124/100,000 people; in urban areas, the prevalence rate is 136/100000 people, in contrast to rural areas, which is 165/100,000 people [4].

 The most specific characteristics of stroke are contra lateral sensory impairments, loss of motor control, difference in muscle tone, muscle weakness, and lack of postural control, and balance. either unilateral or contralateral side of the body exhibiting numbness or weakness in the facial region, upper or lower extremity has been identified as the clinical syndrome of stroke. loss of coordination, dizziness, and the inability to move. One of the key predictive factors for stroke patients' functional outcomes is trunk control and gait performance. Therefore, one of the primary objectives of stroke rehabilitation is to regain stability in the trunk and gait functionality. Immediately following a stroke, reduced skeletal muscle activity, decreased contractility, and decreased pelvic motions are prevalent, as is trunk control impairment. Numerous research investigations have employed diagonal pattern training for trunk muscle control retraining that assists stroke patients move their pelvis and spine in specific ways. A particular method for trunk rehabilitation training is diagonal pattern training, which enhances trunk asymmetry, flexibility, and strength given by different planes [5]. The common parameters that are used to evaluate the gait control and gait are the Trunk Impairment Scale (TIS) and the Timed Up and Go (TUG) test. Numerous physiotherapy managements for stroke are currently in practice, which include core strengthening exercises, stair gait training, and constraint-induced therapy, contributing to the enhancement of trunk stability and improvement of gait patterns in stroke. The contributing factors of impaired trunk control encompass dysfunction, movement techniques, and biomechanical structure-related issues. A key component for sustaining body stability in both calm posture and sudden postural disruption is proprioception [6].

Stroke-induced hemiplegia significantly impairs trunk stability and lower limb function, necessitating targeted rehabilitation strategies to restore motor control and functional capacity. The study addresses the critical need for effective rehabilitation interventions tailored to stroke hemiplegic patients, who often struggle with impaired trunk stability and lower limb function, and seeks to assess the efficacy of a diagonal pattern exercise program in enhancing trunk and lower limb function in patients with stroke-induced hemiplegia. The primary objective is to determine whether this specialized exercise regimen can facilitate neuromuscular re-education and improve the coordination of trunk and limb movements, thereby contributing to superior functional recovery and an enhanced quality of life for these patients. Given the growing recognition of the importance of trunk control in overall functional recovery, there is a pressing need to explore and validate exercise programs that specifically target these areas. This study aims to fill this gap by investigating the potential of a diagonal pattern exercise program to enhance motor function, thereby contributing to more effective and targeted rehabilitation strategies for stroke hemiplegic patients.

## Materials and methods

Selection of subjects

This experimental approach involved an initial and final test framework and this study was conducted at the Outpatient Department of Madha College of Physiotherapy, Chennai. Informed consent and ethical approval were obtained from the Institutional Ethical Committee (ABV-22/P-MCP/PHYSIO/IRB/@2022-2023). This study employed a single-group design using a convenience sampling method to select 20 stroke patients who met the inclusion criteria, including having suffered a stroke more than 6 months prior, being aged between 45 and 70 years, having a Mini-Mental State Examination score of 24 or higher, the ability to maintain an independent standing posture for at least 30 seconds, and the ability to walk more than 30 meters without assistance. Peripheral vascular disorders, coronary circulation disorders, cerebellar ataxia, cardiopulmonary complications, and previous history of surgical procedures on the spine were among the exclusion criteria. The study spanned 6 months, during which a treatment program consisting of 20 sessions over four weeks was implemented. Independent variables included diagonal pattern exercise for the experimental group and single-plane training for the control group, while dependent variables were quantified using the TIS and the TUG test to measure trunk control and gait, respectively. Required materials included a height-adjustable chair, a stopwatch, a measured 3-meter distance, and an assessment scale.

Procedure

After a comprehensive explanation of the study protocols and obtaining consent from each participant, 20 stroke subjects who met the predefined inclusion criteria were randomly assigned to two equal groups of 10: Group A, the experimental group receiving diagonal pattern exercises for the trunk and lower limbs, and Group B, the control group undergoing single-plane training. Before the intervention, all subjects underwent pretest assessments for trunk control and gait. Over four consecutive weeks, both groups received treatment sessions lasting 45 minutes, conducted five days per week. Post-interventional assessment scores were recorded following the completion of the intervention period. Outcome measures for trunk control and gait were clearly defined and collected using standardized assessment tools.

Intervention

Diagonal Pattern Exercise for Group A

The experimental group was administered with diagonal pattern exercise for the trunk and lower limb, which is a therapeutic technique for trunk and lower limb rehabilitation designed to overcome musculoskeletal movement disorders. The workout was done while seated on a bed with a height adjustability. The subjects were given clear instructions to inter-clasp their fingers together to hold the affected arm with the support of the unaffected arm to move the paralyzed hand. A total of five phases to the diagonal pattern exercises, along with two diagonal patterns have been included in each stage. Each workout was done five times in a minute, with an interruption of thirty seconds in between. A set of diagonal pattern exercises was deemed complete when five distinct exercises were completed in the manner previously mentioned. The full 30 minutes were allocated to three sets, with a 100-second break between each set. During the exercises, all participants were instructed to fix their gaze on the points of the clasped hands [7].

Single-Plane Training Protocol for Group B

The control group was administered with single-plane training protocol focusing on bodyweight exercises for trunk flexion and extension, hip flexion and extension, knee flexion and extension, and ankle dorsiflexion and plantarflexion. Trunk exercises included seated and standing movements aimed at improving core stability and range of motion. Hip exercises involved controlled movements to strengthen hip flexors and extensors. The knee exercises included seated or standing flexion and extension to enhance quadriceps and hamstring strength. Body weight ankle exercises targeted dorsiflexion and plantarflexion to improve lower leg muscle control, which is essential for walking and weight-bearing activities. All exercises were performed uniformly for 20 repetitions per set, across three sets, within a brief 30-minute exercise session [8].

Outcome measures 

The TIS demonstrates strong reliability, with high kappa and weighted kappa values indicating substantial agreement between raters on item-by-item assessments. Intraclass correlations (ICCs) for subscale scores and total scores show excellent consistency over time and between observers. Test-retest reliability is robust (ICC = 0.96), as is interobserver reliability (ICC = 0.99). These findings, coupled with high percentages of agreement and acceptable measurement error, underscore the TIS's reliability for clinical use and stroke research, supporting its utility in assessing trunk control and guiding treatment decisions effectively [9].

The TUG test's profound reliability across multiple tests in senior citizens with persistent stroke underscores its efficacy in consistently measuring functional mobility. It effectively distinguishes between healthy elderly individuals and stroke patients, highlighting its utility as a sensitive tool for assessing mobility impairments. These findings reinforce the TUG test's reliability and its role in evaluating functional mobility and gait performance in stroke rehabilitation contexts [10].

## Results

The participants were evaluated employing the aforementioned statistical instruments. The student's *t*-test was implemented for evaluating the data. The prior and following test values for Groups A and Group B were contrasted using the paired *t*-test. Group A and Group B's group computation was estimated using the unpaired t-test and the Berg balance and fall effectiveness scales.

The before-testing averages of the groups participating in the experiment and the control demonstrated no discernible variation between them in the meantime, emphasizing that although they were chosen from the identical population, the two groups have no correspondence with individuals doing distinct exercise regimens. Consequently, this allows the null hypothesis to be disproved. The trunk impairment scale and the TUG test showed a significant improvement in the control group when the pre- and posttest values were evaluated at the 5% level of significance (Tables 1-2). 

**Table 1:** Statistical results of the Trunk Impairment Scale obtained in Groups A and B. The table presents the analysis of the Trunk Impairment Scale for Group A and Group B. For Group A, the paired *t*-test, with 9 degrees of freedom and a significance level of 5%, yielded a calculated t-value of 11.0457, which is greater than the tabulated value of 2.262. This result indicates a significant difference between the pretest and posttest values. For Group B, the paired *t*-test, with 9 degrees of freedom and a significance level of 5%, yielded a calculated *t*-value of 9.3915, which is greater than the tabulated value of 2.262. This result also shows a significant difference between the pretest and posttest values. Additionally, the unpaired *t*-test, with 18 degrees of freedom and a significance level of 5%, yielded a calculated *t*-value of 3.7185, which exceeds the tabulated value of 1.734. This result indicates a significant difference between the posttest values of Group A and Group B.

Group	Pretest mean	Standard deviation	Unpaired t-test	Posttest mean	Standard deviation	Unpaired t-test	Paired t-test
A	9.20	±1.75	3.0137	17.40	±1.43	3.7185	11.0457
B	11.30	±1.34		9.391	±1.69		9.3915

**Table 2:** Statistical results of the Timed Up and Go test obtained in Group A and Group B. This table presents the analysis of the Timed Up and Go test for Group A and Group B. For Group A, the paired *t*-test, with 9 degrees of freedom and a significance level of 5%, yielded a calculated *t*-value of 15.076, which is greater than the tabulated value of 2.262. This result indicates a significant difference between the pretest and posttest values. For Group B, the paired *t*-test, with 9 degrees of freedom and a significance level of 5%, yielded a calculated *t*-value of 9.397, which is greater than the tabulated value of 2.262. This result also shows a significant difference between the pretest and posttest values. Additionally, the unpaired *t*-test, with 18 degrees of freedom and a significance level of 5%, yielded a calculated *t*-value of 5.035, which exceeds the tabulated value of 1.734. This result indicates a significant difference between the posttest values of Group A and Group B.

Group	Pretest mean	Standard deviation	Unpaired *t*-test	Posttest mean	Standard deviation	Unpaired *t*-test	Paired *t*-test
A	27.70	±2.35	2.1168	13.97	±1.89	5.035	15.076
B	25.32	±2.69		17.96	±1.62		9.397

The result of this study regarding the significant improvement of trunk control in the experimental group to the control group might be explained by several mechanisms such as the coordinated contributions of multiple physiological sub-systems. The first explanation is related to the role of diagonal pattern exercise in improving muscle activity around the trunk.

## Discussion

Trunk control deficiencies complying with a cerebrovascular accident entail functional disability in survivors and a rise in caregiver dependence on them. The primary objective of this study was to ascertain the extent to which diagonal pattern training for both the trunk and lower extremities might enhance trunk control and locomotion following a stroke, as evidenced by the TIS and the timed up-and-go test.

In a research done by Patil et al. in 2019, the results revealed that trunk proprioceptive neuromuscular facilitation incorporated alongside conventional functional mobility exercises was more constructive than utilizing conventional functional mobility tasks solely for boosting functional mobility in hemiplegics and for early trunk facilitation [11]. Concomitantly a parallel study by Shanmugananth et al. in 2015 indicated that proprioceptive neuromuscular facilitation (PNF) procedures targeting both the upper and lower trunks were pertinent for aiding hemiplegic individuals regain their control over their posture. More specifically, hemiplegic patients' posture regulation was enhanced more through the lower trunk PNF approach than the upper trunk PNF technique. These findings indicate that strengthening the stability of posture in those individuals could potentially be more feasible when concentrating on the lower trunk [12]. Similarly, in research by Hazarikahe et al. in 2022, the results revealed that subjects in Group A, who received the PNF technique, exhibited greater effectiveness compared to Group B, particularly in trunk facilitation with specific movement patterns, resistance, and stretching. Consequently, the subjects in Group A showed remarkable improvement in trunk stability in contrast to those in Group B who received trunk NDT [13].

Altaim et al. in 2023 showed that the exercises, including pelvic clock and static bicycle, positively influenced gait and trunk functions in chronic hemiplegic patients. Our findings highlight significant improvements in various components of the Walking Gait Scale (WGS), such as handheld gait aid usage, unaffected side step length, guardedness, mid-swing circumduction, and pelvic rotation in terminal swing. Integrating WGS alongside other functional assessment scales provides comprehensive insights into gait standards and deviations. It effectively captures body posture across gait phases, revealing pathological patterns adopted by patients during walking. Similar improvements were observed in the TIS, particularly in static sitting, dynamic sitting, and coordination, further supporting its utility in assessing trunk function during recovery [14].

Group A received diagonal pattern exercises for the trunk and lower limbs, while Group B received single-plane training for 4 weeks. At the end of this period, participants underwent evaluations using the aforementioned outcome measures. The individuals being studied were simultaneously involved in trunk bending, lateral bending, and rotational motions throughout diagonal patterning movements. This approach broadened the subjects' versatility of motion and engaged additional muscle groups. Significantly, the diagonally patterned movements employed in our study incorporate repetitive lifting and chopping motions that are based on PNF techniques. The PNF's cutting and lifting methodology drastically raised the stroke patients' trunk disabilities scale in an earlier investigation. Considering that these outcomes support our findings, diagonal pattern training has been reported to be more effective than single-plane training. The results of this study highlight the effectiveness of a diagonal pattern exercise program in enhancing trunk stability and lower limb function in stroke hemiplegic patients. The significant improvements observed in the experimental group suggest that this specialized exercise regimen successfully addresses the complex neuromuscular deficits associated with hemiplegia. These findings are consistent with existing literature, which emphasizes the importance of trunk control in overall functional recovery post-stroke. The study underscores the potential of targeted rehabilitation strategies, such as diagonal pattern exercises, to facilitate meaningful improvements in motor function, ultimately contributing to better patient outcomes.

Limitations of the study

The limited sample size could constrain the extent to which these conclusions can be implemented. This study was conducted as a short-term surveillance, restricting the determination of long-term impacts of the therapies. 

Recommendations for the study

A substantial number of samples can be utilized to show how an intervention is effective. The trunk control of cerebrovascular accident patients can be evaluated by the investigations employing supplementary outcome measures. It is possible to do a long-term follow-up to ascertain the impact of the intervention.

## Conclusions

The group performing the experiment's diagonal pattern exercise outperformed the control group's single-plane training, as demonstrated by the results. The study demonstrated that a four-week diagonal pattern exercise program significantly improves trunk control and lower limb function in stroke-induced hemiplegic patients. The experimental group showed a marked improvement in trunk stability and motor coordination compared to the control group, as evidenced by statistically significant results (*P* < 0.05) on the TIS and TUG tests. These findings validate the efficacy of the diagonal pattern exercise program in facilitating neuromuscular re-education and enhancing functional recovery, thereby supporting its use as a targeted rehabilitation strategy for improving quality of life in stroke hemiplegic patients. Overall, the study underscores the potential of incorporating diagonal pattern exercises into rehabilitation programs to achieve superior functional gains in stroke patients. The findings advocate for integrating this evidence-based, cost-effective strategy into clinical practice to enhance the quality of life and improve recovery trajectories for individuals with persistent stroke-related impairments.
